# Chimpanzees’ (*Pan troglodytes*) problem-solving skills are influenced by housing facility and captive care duration

**DOI:** 10.7717/peerj.10263

**Published:** 2020-11-25

**Authors:** Sofia Forss, Alba Motes-Rodrigo, Christine Hrubesch, Claudio Tennie

**Affiliations:** 1 Department of Early Prehistory and Quaternary Ecology, Eberhard-Karls-Universität Tübingen, Tübingen, Germany; 2 Department of Anthropology, University of Zürich, Leintalzoo, Schwaigern, Germany

**Keywords:** Physical cognition, Chimpanzees, Housing facility, Experience effect, Problem-solving skills

## Abstract

Although a large body of primate cognition research is done in captive institutions, little is known about how much individuals from different facilities vary in their experiences and cognitive skills. Here we present the results of an experimental study investigating how *physical cognitive skills* vary between chimpanzees in relation to captive settings and their time in captivity. We tested 59 chimpanzees housed at two different captive facilities (a rehabilitation center (sanctuary) and a zoo) in three problem-solving tasks. Our results showed that chimpanzees at the two housing facilities significantly differed in overall task performance. On average, the sanctuary chimpanzees outperformed the chimpanzees housed at the zoo in the detour reaching task and the honey trap task. However, the zoo chimpanzees performed slightly better on average in the learning task. We propose that, for this particular sample, the documented differences result from a combination of factors, such as prior experience with cognitive testing, motivation levels and varying degrees of human exposure. Within the sanctuary sample, we found that chimpanzees who arrived at an earlier age at the sanctuary and had therefore spent a larger percentage of their lives in a captive environment, were better problem-solvers than those that arrived at a later age to the sanctuary. Thus, rehabilitation and time in captivity contributed to improved physical cognitive skills in sanctuary chimpanzees. Our results highlight the importance of studying intraspecific variation and the effect that previous experience and living conditions might have on physical cognitive skills in non-human apes. Accordingly, we should be cautious when extrapolating findings of cognitive studies from one population to the species as a whole.

## Introduction

In order to understand the evolution of cognitive abilities we need to disentangle environmental and genetic influences from cognitive phenotypes in non-human animals. To this end, it is important to consider intraspecific variation and to identify what factors correlate with between-individual differences (Boesch, 2020). Previous research has shown that early life experience can influence cognition in several species: enriched captive conditions during early life increase spatial learning abilities in fish (*Salmo salar*) (Salvanes et al., 2013); the early incubation environment of lizard eggs (*Pogona vitticeps*) influences the adult lizards’ socio-cognitive skills assessed through social learning tasks (Siviter et al., 2017); early maternal separation reduces learning ability in male mice (*Mus musculus*) (Wang, Jiao & Dulawa, 2011) and hand-raised parakeets (*Melopsittacus undulatus*) perform better at object permanence than parent-raised ones (Funk, 1996).

In humans (*Homo sapiens*), part of the observed intraspecific variation in cognitive abilities is explained by genetic inheritance (Deary, Johnson & Houlihan, 2009; Nisbett et al., 2012; Bates, Lewis & Weiss, 2013). Thus, to identify the variation in cognitive abilities explained by environmental factors and early-life experiences in our species, large research efforts have been deployed into studies of identical twins and adopted children. Such study design has provided insight into how, besides genes, the nurturing environment where a child is raised contributes to outcomes such as educational attainment and income later in life (Sacerdote, 2011). Compared to children from high socio-economic-status (SES) families, children as young as six months old from low SES families already show lowered attentiveness influencing a cascade of cognitive skills (Clearfield & Niman, 2012). Furthermore, studies on adopted institutionalized children have shown that children who move to foster families at an earlier stage of development have better chances at cognitive recovery from early social deprivation (Nelson et al., 2007).

Similar to humans, studies on chimpanzees suggest that great ape (henceforth apes) cognition is influenced both by genetics (Hopkins, Russell & Schaeffer, 2014) and the socio-cultural environment where the individuals develop (Russon, Bard & Parker, 1998; Reader & Laland, 2002; Russell et al., 2011; van Schaik & Burkart, 2011). Ideally, we would assess the influence of experience and early life environments on cognitive abilities in the species’ ecological context. However, it is often challenging to conduct controlled, cognitive experiments in the natural environment of apes. Due to this limitation, most cognitive studies are performed in captive, settings where the learning opportunities during experiments can be controlled for.

Captive apes show large individual variation in cognitive performance (Herrmann & Call, 2012) and even within the same facility, chimpanzees have been shown to differ in their tendencies to use social information during problem-solving tasks (Watson et al., 2018). Thus, when measuring cognitive skills in captive apes, we need to take into consideration this variation as well as its potential underlying factors. Sources of intraspecific variation can be different motivational levels among individuals to participate in an experiment; familiarity with the test apparatuses and methodological procedures and differences in housing conditions or routines between facilities. In addition, the apes’ contact with peers, their degree of human contact and their exposure to human artefacts can also influence the apes’ performance in cognitive tasks (Tomasello, Savage-Rumbaugh & Kruger, 1993; Call & Tomasello, 1996; Tomasello & Call, 2004; Bering, 2004; Furlong, Boose & Boysen, 2008; Damerius et al., 2017a; Damerius et al., 2017b). Lastly, the rearing background where an individual develops is also likely to influence the individual’s cognitive development. For instance, enculturated apes (defined as apes raised by humans in an anthropomorphic environment and attended to as intentional agents exposed to a wide variety of human cultural experiences; Tomasello, Savage-Rumbaugh & Kruger, 1993) show enhanced physical cognitive skills compared to conspecifics reared by their own mother or nursery-reared with peers (Russell et al., 2011).

Therefore, research on ape cognition would benefit from a better understanding of the extent of the variation *between* captive populations, namely whether chimpanzees at different facilities vary in their physical cognitive skills. In addition, studies exploring the role that different factors play in ape performance within a housing facility can further improve our knowledge regarding sources of intraspecific variation. If, for instance, housing conditions or the duration of human care influence apes’ performance in cognitive tasks, research performed at a single location should not be automatically extrapolated to other populations of the same species or to the species as a whole (Boesch, 2020).

In the present study we systematically compared the problem-solving skills of chimpanzees housed at two different facilities (a zoo and a sanctuary) in several cognitive tasks in order to assess possible between-facility differences. As we were also interested in the individual variation within a single facility, we used the sanctuary sample to evaluate if problem-solving skills differed depending on an individual’s age at arrival at the sanctuary and on the cumulative time they had spent in captive care.

## Methods

### Ethical statement

All problem-solving tests were non-invasive and solely behavioral observations were made. All tests complied with the ethical principles set by the UWA, Ugandan Wildlife Authority (UWA/COD/95/06) and the National Council for Science and Technology (UNCST) (reference number NS27ES). The study was also supported by the BIAZA Animal Care Committee (British and Irish association for Zoos and Aquariums) and approved by the Swiss veterinary institution (2960/ 29815). Barbara Gessmann (Leintal zoo, Schweigern, Germany) provided permission to test the chimpanzees at Leintal zoo.

### Subjects and facilities

We tested chimpanzees’ problem-solving skills with three tasks targeting different aspects of physical cognition that yielded six different cognitive measurements (Table 1). The total sample size varied for each task from *N* = 49 to *N* = 59, as subjects participated on a voluntarily basis in the tasks (Table 1). We collected part of the data (*N* = 40) at Ngamba Island chimpanzee sanctuary in Uganda during September and October 2017. For data collection at Ngamba Island, a field permit was granted from the Chimpanzee Sanctuary and Wildlife Conservation Trust. At the time of data collection, with the exception of two chimpanzees born at the institution (not part of our study), the sanctuary housed a large group of orphan and confiscated chimpanzees, most of them rescued from the illegal pet and bushmeat trade. These chimpanzees have gone through rehabilitation due to traumatic experiences including maternal loss, malnutrition, and periods of restricted movement in small cages. They are currently housed and taken care of by the chimpanzee sanctuary and conservation trust in a semi-natural environment. At Ngamba Island the chimpanzees have access to 95 acres of tropical forest but can return to the facilities voluntarily for feeding multiple times each day as well as for overnighting. The chimpanzees at Ngamba are familiar with being individually separated occasionally for health checkups or research purposes. The sanctuary provided an informative record on the background histories of some of the chimpanzees, including their age at arrival at the sanctuary, health condition upon arrival at the sanctuary and what kind of environment they had been in prior to rescue (i.e., found in cage, malnourished, human held as pet/ “entertainment hostage” or brought straight to sanctuary, described in Table S1). This set of information enabled us to address (within the Ngamba Island sanctuary) how the age at arrival at the sanctuary and the percentage of lifetime spent at the sanctuary influenced task performance.

**Table 1:** Overview of problem-solving tasks, cognitive measurements, and performance.

**Problem-solving task**	**Cognitive measurement**	**NNgamba**	**NLeinthal**	**Success at Ngamba**	**Success at Leinthal**
Detour reaching	Find solution (Yes/No)	40	19	98%	47%
Detour reaching	Find solution without physical exploration (Yes/No)	40	19	63%	0%
Visible honey trap	Find stick solution (Yes/No)	40	14	95%	62%
Visible honey trap	Innovate rope solution (Yes/No)	40	14	38%	0%
Reversal learning	Learn color-food association (Yes/No)	35	14	77%	100%
Reversal learning	Learn the reverse pattern (Yes/No)	35	14	54%	43%

**Notes.**

a % of chimpanzees that correctly solved the task.

Our second sample was comprised by the chimpanzees housed in Leintal zoo, Germany, during September and October 2017. With a total group size of 33 individuals, this group of chimpanzees represents the largest zoo housed chimpanzee community in Europe. Except for a few training experiments (observational data, Hrubesch 2007–2008), this chimpanzee community had not been targeted for research projects on cognition before the data was collected for this study. The majority of individuals at Leintal zoo were zoo born and mother-reared, with the exception of six individuals, one with an unknown background and five that had lived with humans up to the age of roughly one year. The rearing information of those chimpanzees that participated in the cognitive tasks is included in Table S1. The chimpanzees at Leintal zoo live in a large outdoor area comprised of multiple enclosures connected to indoor quarters with attached sleeping rooms. Given that the chimpanzees at Leintal zoo were not used to being separated from their group (except for health check-ups) and because they could participate in our cognitive tasks on a voluntarily basis, our zoo sample varies in size between 14 and 19 individuals depending on the task. Due to the large peer group constellation and scarce experience with cognitive tests (including materials and apparatuses), the enculturation of these chimpanzees may be described as minimal for captivity (with the exception of the five hand-reared individuals).

### Cognitive tasks and experimental set up

We tested the chimpanzees in three novel (to them) problem-solving tasks (detour reaching task, visible honey trap task and reversal learning task) generating six different measurements on different aspects of physical cognition (Table 1). The tasks were performed in the above listed order, to ensure that all subjects participating in the study had a similar experience through the testing phase. These standardized problem-solving tasks have been previously used to investigate physical cognition in orangutans (Forss et al., 2016; Damerius et al., 2017a; Damerius et al., 2017b). Prior to testing the chimpanzees in the problem-solving tasks, we also assessed the level of human orientation of each of the chimpanzees through a previously established Human Orientation Index, HOI (Damerius et al., 2017a). In each task, every subject was tested alone and thus for a shorter period separated from its social group. Throughout the study, all chimpanzees were fed according to their normal daily routines and none of the subjects were food deprived during the experiments.

At Ngamba Island, the chimpanzees were tested in their sleeping quarters after the morning feeding before they went into the forest habitat. At Leintal zoo testing took place in the smaller sleeping rooms mostly before mid-day. All tests were recorded with SONY HD handy cameras.

Each subject was tested once on each task (meaning that each problem-solving task was presented only one time to each subject) with the exception of the reversal learning task, which measures working memory and therefore was presented to each subject on four consecutive days. For each task, the subjects’ response towards the test apparatus was coded from video recordings. There were no demonstrations or pre-training trials.

### Detour reaching task

**Figure 1:**
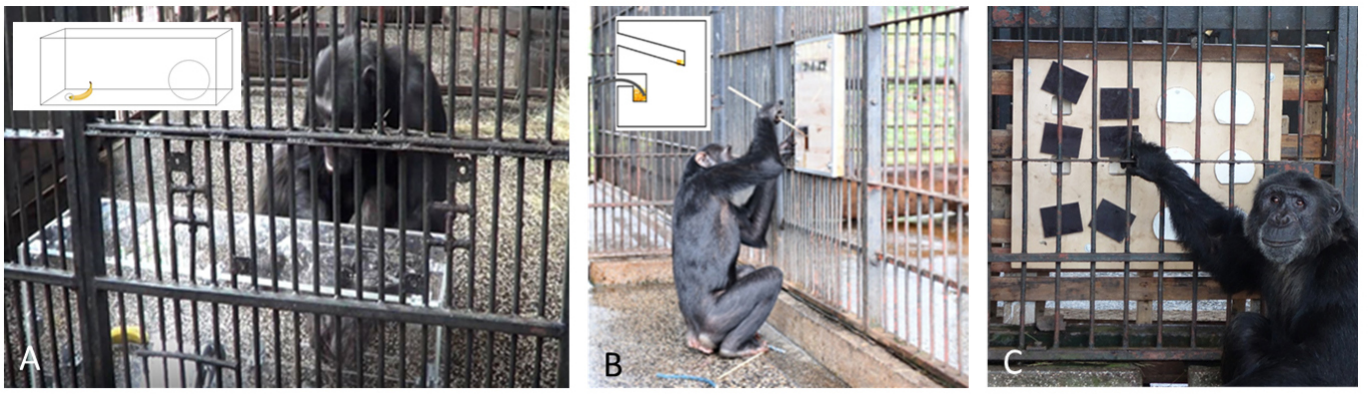
Test apparatuses for assessing problem-solving skills. (A) Detour reaching task, (B) visible honey trap and (C) reversal learning task.

The detour reaching task tests for inhibitory control (Fig. 1A). In the task, the subject is allowed access to a large, completely transparent, Plexiglas box (100 cm × 30 cm × 30 cm). The front side of the box has one small round hole within a 50 cm distance of a larger rectangular hole where the subjects’ arm can pass through. Inside the box, a food reward (whole banana) is placed behind the small hole. Each subject is given a maximum of five minutes to solve the task. The variables measured in this task that were later used as dependent variables in the statistical models were (a) whether the chimpanzee solved the task and reached for the banana through the big hole (Yes/No) and (b) if the chimpanzee managed to solve the task without any physical trial and error exploration of the box (Yes/No). Explorative actions were defined as touching the Plexiglas box, poking with a finger through the small or big holes, hitting, lifting, or kicking the box.

### Visible honey trap task

The visible honey trap task (henceforth honey trap task, Fig. 1B) measures the understanding of tool properties of two types of tools: a rigid stick and a bendable rope. The upper part of the apparatus contains a straight channel that fits a 40 cm long stick. This channel was baited with a stick dipped in a little bit of honey. At the lower part of the trap there is a curved channel filled with much more honey to motivate the subjects to attempt this more difficult problem requiring the innovation of a novel tool (in this case a rope fragment laid out on the floor) to dip for honey. As the stick does not fit in the curved channel, the subjects need to dip a bendable piece of rope into the curved channel to extract the honey. The front side of the apparatus is transparent so that the subjects can see the different shapes of the channels as well as the honey. Each subject is provided with two extra sticks and three pieces of rope on the floor in front of the apparatus. Each trial lasts ten minutes during which the individuals have time to solve both tool tasks. As dependent variables for the statistical analyses we measured (a) if a subject solved the stick task by either re-using the inserted stick or inserting one of the provided sticks into the straight channel (Yes/No) and (b) if a subject solved the rope task by using the rope to obtain honey from the curved channel (Yes/No). We defined a subject as successful when he/she inserted 1/3 of the length of the stick in the straight channel or 1/3 of the rope in the bent channel. Given that this test apparatus was presented to the chimpanzees within their enclosure and they could interact with it for a longer time, we also assessed their motivation to interact with the apparatus, measured as time the chimpanzees spent exploring the apparatus before they found the rope solution or until end of task.

### Reversal learning task

The reversal learning task measures working memory, inhibition, and flexibility (Fig. 1C). In this task the subjects need to first learn the association between the color of six lids that hide a food reward (black or white) and distinguish those lids from the other color that covers empty holes. A subject is considered to have learned the right color association when, out of the first six lids that he/she touches, five are of the correct/baited color. After learning the color-food association, the subjects need to pass a control trial fulfilling the same criterion. Once this control trial was conducted, we switched the color that hides the food rewards and thereafter the subject needed to learn the reverse pattern. Each individual was tested in this task for four continuous days and subjects were tested in three trials every day for four continuous days. However, if a subject learned the color association in the third trial of a day, we ran the additional control trial that day to confirm that the subject had learned the correct color-food association. In this task we measured (a) if the subjects learnt the correct color-food association (dependent variable) and (b) if the subjects learnt the reverse-food color association (dependent variable).

### Human Orientation Index

To quantify variation in human habituation we performed an additional test that measured each ape’s Human Orientation Index (HOI) previously established by Damerius et al. (2017a). The HOI differs from the standard Human Intruder Test (HIT) (Gottlieb & Capitanio, 2013) because it does not incorporate any threatening positions or movements, instead it aims at testing reactions towards a stranger with and without food. We calculated the HOI of each chimpanzee by testing them individually in three subsequent experimental conditions. In the first condition a male human (to be consistent between facilities) who is unfamiliar to the apes, calmly approaches the enclosure and positions himself 1–1.5 m in front of the enclosure and stands still for 30 s with the side of his body towards the enclosure. In the second condition, the man turns around so that his body frontally faces the subject and tries to establish eye contact with the subject for one minute. Because we additionally wanted to quantify the orientation to humans when food is involved, and when it is not, we conducted one additional condition to those implemented by Damerius et al. (2017a) including an unfamiliar human offering food out of reach of the chimpanzee. In the third condition, the man remains frontally facing the ape but instead of keeping eye contact with the subject, he directs his gaze down at the ground and takes out a preferred food item (peanuts) from his pocket and holds the food beside his body in his hand, out of reach of the subject for 30 s. After condition three is completed, the food reward is placed in front of the enclosure and the test subject can collect it. For each condition we coded the proximity of the ape to the man as follows: 0 = the ape positioned itself as far away as possible from the human; 1 = the ape was more than one meter away from the human; 2 = the ape was within one meter from the human; 3 = the ape placed itself as close to the human as possible. We also scored the subject’s initial behavioral reaction in each condition as follows: 0 = a negative reaction, including retreat, stress vocalization, pilo-erection, nervous swinging or turning away from the human; 1 = a neutral reaction, including resting, moving calmly or play behavior; 2 = a positive reaction, if the ape approached the human; 3 = an active positive reaction including begging gestures (either by using lips or hands) or any other proactive behavior aimed at establishing contact with the human as well as attempts to trade objects from the enclosure for food. In addition, for each condition we scored whether or not the ape engaged in any active positive reactions (begging, trying to get the man’s attention) beyond the initial coded behaviors. The sum of the proximity and reaction scores across conditions constitutes the Human Orientation Index (HOI), which can range from 0 to 21.

### Statistical analysis

We conducted the statistical analysis in R (version 3.3.3, R Core Team, 2017) and RStudio (version 1.1.383). We z-transformed covariates (age, age at arrival at the sanctuary and percentage of life at sanctuary) to a mean of 0 and standard deviation of 1 before including them in the models to facilitate the interpretation of the coefficient estimates (Schielzeth, 2010). Factors (facility and sex) were manually dummy coded before being introduced in the models. All data and code employed in these analyses can be found in the link https://osf.io/qeydu/.

#### Comparisons between facilities in task performance

To investigate if there were differences in the cognitive measurements between facilities, we fitted a Generalized Linear Mixed Model (glmm) with binomial error structure and logit link function (Lee & Nelder, 1998). The response in Model 1 was a binary variable composed of 1s and 0s with each row indicating the success or failure of an individual in each task. We fitted a single model with a response variable combining the outcomes of all tasks tested in order to reduce the number of models fitted and consequently, the risk of false positives (type I error). As fixed effects, Model 1 included facility, sex and age of the individuals at the time of testing (the last two as control predictors). To prevent pseudoreplication due to the participation of the individuals in several (or all) tasks, we also included the random intercept of individual ID. We adopted a maximal random slope structure (Barr et al., 2013) by including the random slopes of sex, facility (both dummy coded) and age within task. As we were interested in the overall differences in task performance between facilities, the variable of interest in Model 1 was the random slope of facility within task. Task was a factor with six levels (each cognitive measurement). We chose to investigate the differences between facilities in performance across tasks as a random slope rather than an interaction in order to make the model independent of the specific tasks employed and generalizable. Model 1 was fitted with the function glmer from the package lme4 (Bates et al., 2015) using the optimizer bobyqa. The fitted model was checked for collinearity using variance inflation factors (VIF function from package car; (Fox & Weisberg, 2019; Fox & Weisberg, 2011), overdispersion and overall stability (see Fig. S1) without finding any issues. Inference was drawn by comparing the full model with maximal random slope structure with a reduced (null) model lacking the slope of interest (facility within task, Forstmeier & Schielzeth, 2011) but containing all other predictors using a likelihood ratio test (test “Chisq” in the R function anova). Following Bolker et al. (2009), the *p* value of the full-null model comparison was divided by 2. R squared was calculated with the function r.squaredGLMM.

Due to the experimental set up and duration of the task, the honey trap allowed us to further compare the motivation of the chimpanzees from the two facilities to interact with the test apparatus (measured as the time spent interacting with the honey trap before innovating the rope solution). We fitted a linear model with the function lm (package lme4) in which the response variable was the time the chimpanzees spent interacting with the honey trap and the test predictor was facility (Model 2). We further controlled for the effects of sex and age. As before, inference was drawn comparing the full model including both control and test predictors with a null model lacking the control predictor (facility) using a likelihood ratio test. When potential influential cases were investigated in Model 2 using the function dffits, it was found that the maximum absolute value was 2, suggesting that influential cases were not an issue. R squared was calculated with the function r2 from the package “performance” (Lüdecke et al., 2020).

Potential differences in the degree of human orientation (HOI) between individuals housed at the two facilities were investigated by fitting a model with the function polr (package MASS; Venables & Ripley, 2002) with HOI as response variable and facility as test predictor (Model 3). As before, age and sex were included as control predictors. Inference was drawn as described for Model 2. Given the results of Model 3, two post-hoc analyses were conducted in order to investigate if the HOI differences between facilities were due to the scores in non-food related conditions (conditions 1 and 2, Model 3.1) or in the food related condition (condition 3, Model 3.2). These models were identical to Model 3 except for the response variable, which was the combined score obtained in conditions 1 and 2 (Model 3.1) or the score obtained in condition 3 (Model 3.2). R squared was calculated with the function r2 from the package “performance” (Lüdecke et al., 2020).

#### Analysis of within facility variation: effects of arrival age and lifetime spent in the sanctuary

The honey trap was the task with the largest sample size that generated the most variability in performance amongst the chimpanzees. Therefore, we used the outcomes of this task (binary response with one row per individual and task) within the sanctuary sample to further investigate intraspecific variation on cognitive performance, while controlling for housing differences (as only one facility was tested). We assessed how the probability of success in the honey trap tasks varied based on how old the chimpanzees were when they first arrived at the sanctuary (Model 4) and the percentage of their lives they had spent in the sanctuary (Model 5). Given that these two variables (percentage of life in sanctuary and age at arrival) were highly correlated (Spearman correlation, rho=-0.91, *p* < 0.001), they had to be included in different models. This decision was taken based on the fact that the inclusion of correlated variables in the same model increases the uncertainty of the estimates derived from the model, increase the probability of type II errors and makes the assessment of the relative importance of individual predictors in the model unreliable (Freckleton, 2011). Models 4 and 5 were glmms with binomial error structure and logit link function (Lee & Nelder, 1998). The response was binary (composed of 0s and 1s) with one row per individual and task variant (solving the task with a rope or a stick). We included as control predictor in both models the age of the individuals when tests were conducted (z-transformed). The test predictor of interest in these models was the interaction between task (factor with two levels) and the age of the individuals when they arrived at the sanctuary (Model 4) or the percentage of life spent in the sanctuary (Model 5). In Models 4 and 5, task only included two levels corresponding to the two variants of the honey trap task (solved with stick or rope). The random intercept of individual ID was included to prevent pseudoreplication, as individuals were tested in both tasks. To avoid convergence problems, these two models were fitted using the function glmmTBM from the package of the same name (Brooks et al., 2017). Collinearity in Models 4 and 5 was checked with the function vif from the package car without finding any issues. Model stability was visually checked creating stability plots (Figs. S2 and S3) with the packages broom.mixed and dotwhisker (Bolker, 2016). Inference was drawn by comparing the full model containing all control and test predictors with a reduced model lacking the interaction of interest by means of a likelihood ratio test (test set to “Chisq” in function anova). R squared was calculated with the function r2 from the package “performance” (Lüdecke et al., 2020).

Furthermore, we checked inter-rater reliability by having a research assistant (MP) independently code the success variable (Yes/No if a subject solved or not solved the task). The second coder re-coded 15% of the trials, which were randomly selected from all video recorded tasks. The two coders reached a Cohen’s Kappa of 0.81 (N_events_ = 41, *p* = 0.001), which represents a substantial to almost perfect level of agreement (Landis & Koch, 1977).

Although we tried to additionally account for the rearing background of the individuals included in Model 1 (whether they were hand reared by humans or reared by their mothers), this variable was strongly dependent on the facility and very unbalanced. In Ngamba Island Sanctuary, more than 90% of the individuals had been human reared. On the other hand, in Leintal zoo more than 70% of the individuals had been raised by their mothers. Consequently, the inclusion of this variable in the models created strong model instability and it was decided to exclude it from the main analysis. However, we conducted an additional explorative model (Supplementary Model 1) where we investigated the effect that rearing background had on chimpanzee cognitive performance (Fig. S4). This model is identical to Model 1 except for the substitution of facility by rearing background. Model estimates and BLUPS can be found in Tables S2 and S3 respectively. As in the previous models, the stability (Fig. S5), collinearity and overdispersion of the Supplementary Model 1 were investigated without finding any issues.

## Results

### Differences between facilities in task performance

**Figure 2:**
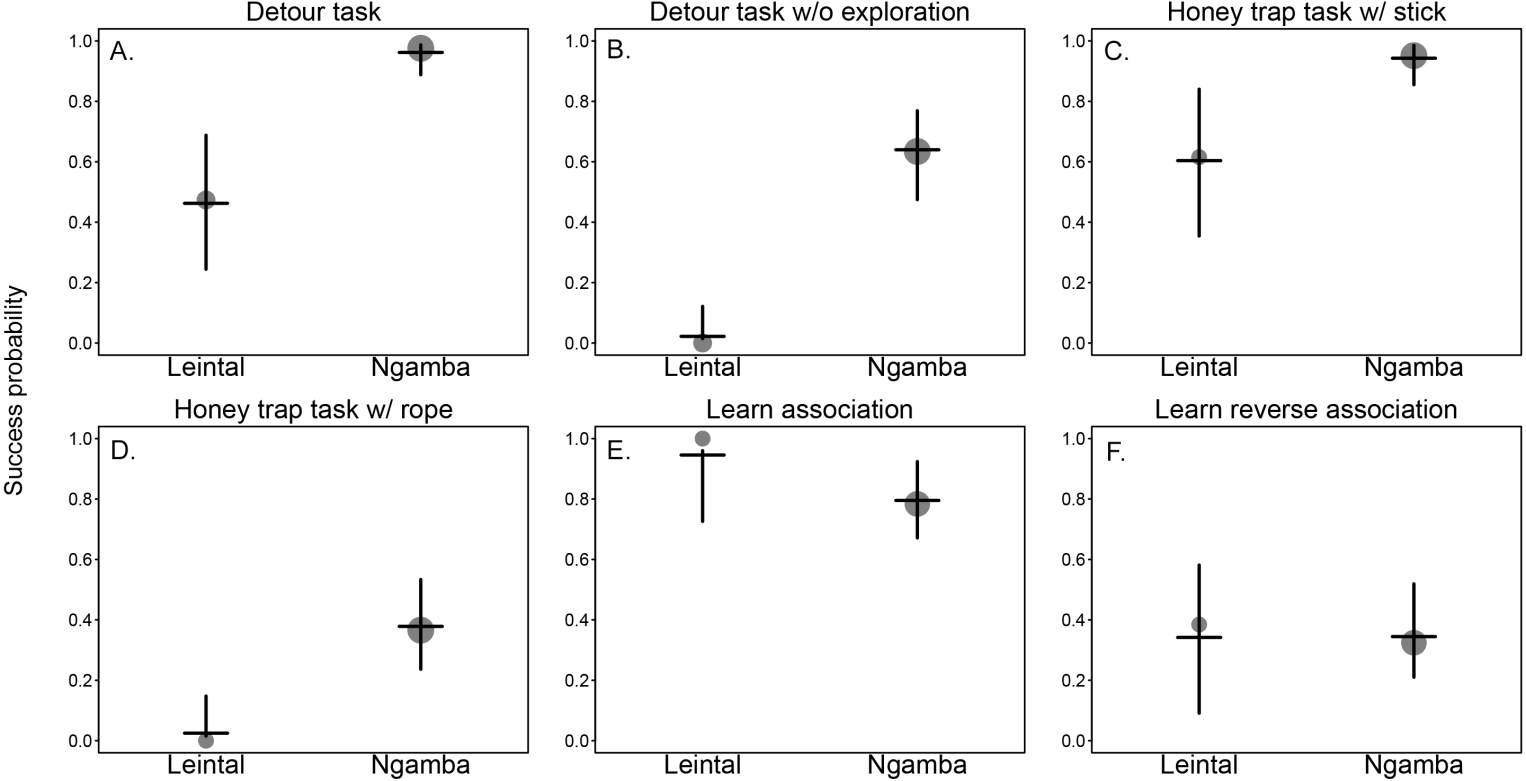
Mean probability of success and 95% confidence intervals across tasks of the chimpanzees housed at Leintal Zoo and Ngamba Island Sanctuary. The area of the dots represents sample size. (A) Detour reaching task. (B) Detour reaching task without exploration. (C) Honey trap task with stick. (D) Honey trap task with rope. (E) color association learning. (F) Reversal learning.

We found that facility had a significant effect on the success probability of the chimpanzees in the different cognitive tasks (Model 1; likelihood ratio test between full and reduced model: *X*^2^ = 21.37, *df* = 1, *p* = 0.001; R^2^ full model=0.57). Table 2 includes the estimates of the fixed effects and Table 3 includes the best linear unbiased prediction (BLUPs) for each of the levels of the random slope of facility within task. The BLUPs (which reveal the variation from the mean slope estimate of facility caused by the different tasks in which the chimpanzees participated) show that in all cognitive measurements from the detour and honey trap tasks, proportionally more chimpanzees at Ngamba Island were successful compared to conspecifics at Leintal zoo (see positive BLUP values in Table 3 and Fig. 2).

The difference between facilities was larger in the more demanding tasks, such as solving the detour reaching without physical exploration (25/40 successful individuals in Ngamba Island versus 0/19 at Leintal zoo) and solving the honey dipping problem with a rope (15/40 successful individuals at Ngamba Island versus 0/14 at Leintal zoo) (Table 1).

In the reversal learning task, the chimpanzees housed at Leintal zoo had an average success probability higher than the Ngamba Island chimpanzees (negative BLUP values in Table 3). This difference was probably driven by the fact that all chimpanzees that participated at Leintal zoo (*N* = 14) learned the first color association (first part of task), but only 27/35 of the chimpanzees at Ngamba Island did so (Table 1, Table 3 and Fig. 2). In the learn-the-reverse part of the task, both facilities had lower proportions of successful chimpanzees compared to the first part of the task (Leinthal zoo: 6/14, Ngamba Island: 10/35, Table 1).

**Table 2:** Fixed effects model estimates, standard errors, degrees of freedom and *p* values of Model 1.

	Estimate	SE	df	*P*
Intercept	−0.987	1.11	^a^	^a^
Facility^b^	2.14	1.15	1	0.06
Sex^c^	0.11	0.20	1	0.70
Age^d^	0.23	0.15	1	0.12

**Notes.**

a Not shown because of having limited interpretation.

b Ngamba Island was the reference category.

c Male was the reference category.

d Age was z-transformed to a mean of 0 and standard deviation of 1. The mean of the original variable was 20.75 and the standard deviation was 7.82.

**Figure 3:**
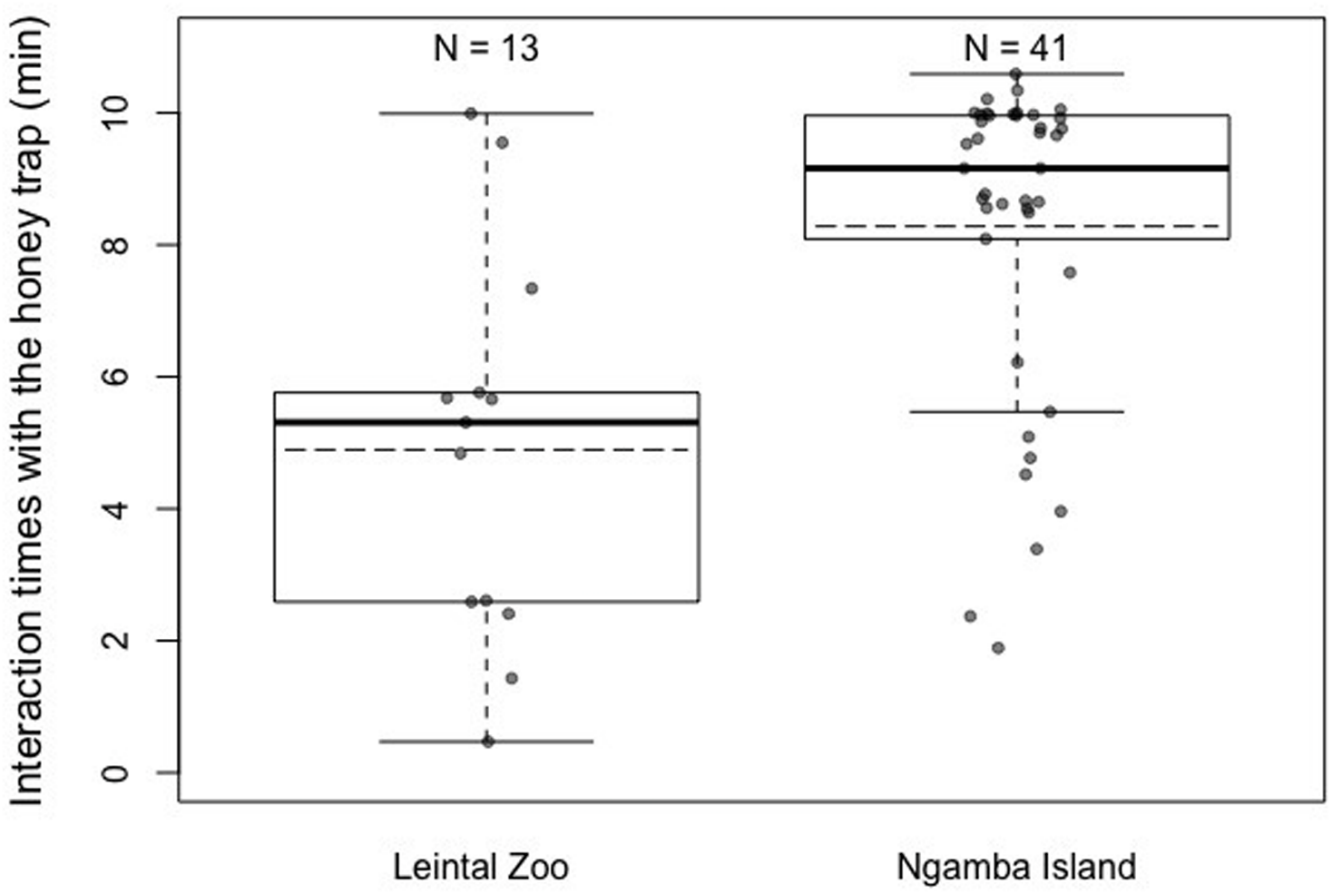
Mean (dashed horizontal lines) and median (solid horizontal lines) interaction times of the chimpanzees housed in Leintal zoo and Ngamba Island with the honey trap. N represents the sample sizes in each of the groups. The top and bottom of the boxes represent the 75th percentile and the 25th percentile respectively. The whisker length was calculated as the percentiles plus or minus 1.5 times the interquartile range.

Regarding differences in motivation levels (measured as time exploring the honey trap task prior to finding the rope solution) between individuals from the two facilities, we found that Ngamba chimpanzees were significantly more motivated to interact with the test apparatus than chimpanzees housed at Leintal Zoo (Model 2; likelihood ratio test between full and reduced model: *X*^2^ = 120.15, *df* = 1, *p* < 0.001; *R*^2^ = 0.26, Fig. 3, Table 4).

**Table 3:** BLUPs of the random effects of Model 1.

	Intercept	Facility^a^
Detour task	1.69	1.24
Detour task without exploration	−1.24	2.23
Honey trap task solved with stick	1.54	0.24
Honey trap task solved with rope	−1.98	1.03
Learn association	1.16	−3.64
Learn reverse association	−1.26	−2.13

**Notes.**

a Dummy coded with Ngamba as reference category.

**Figure 4:**
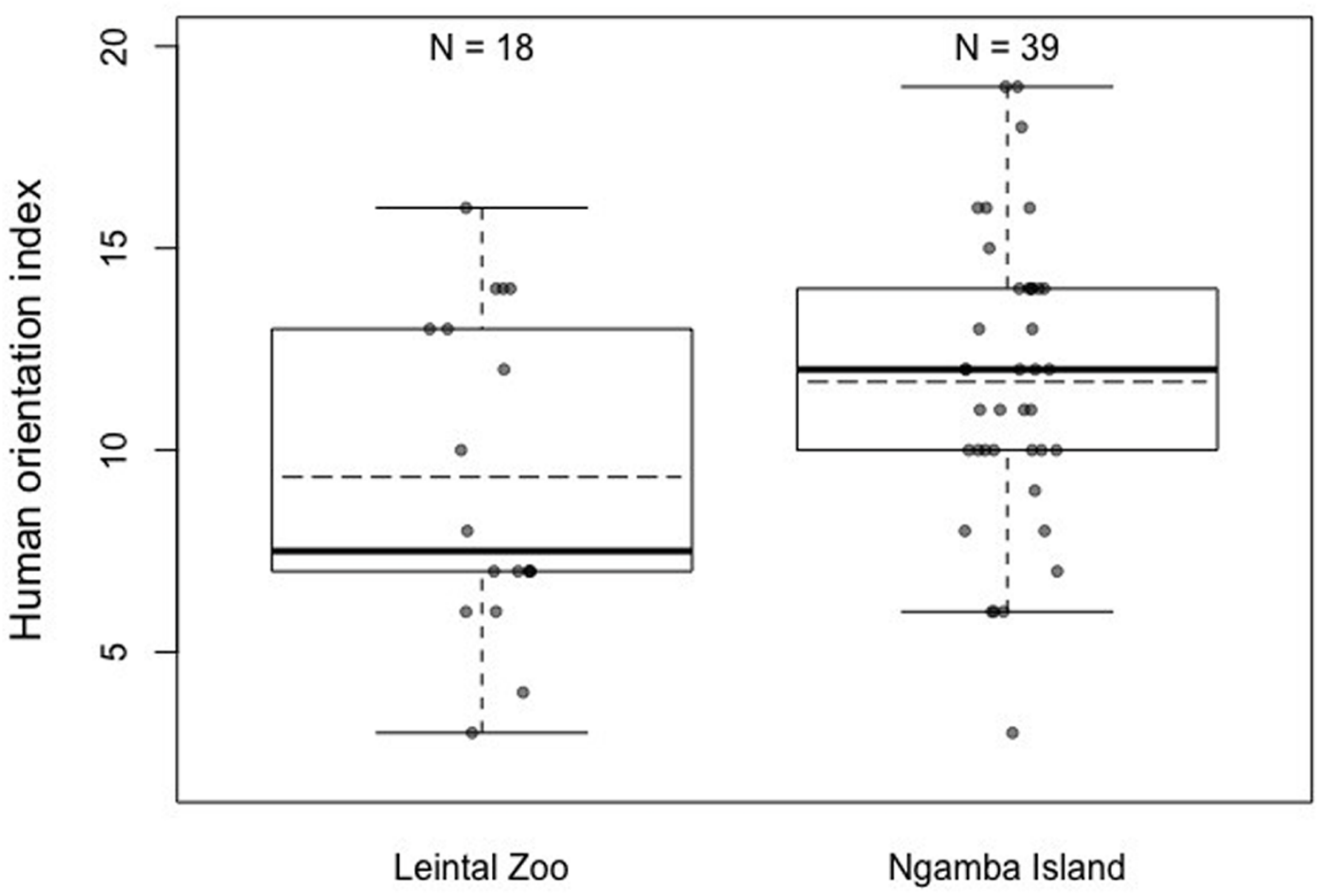
Mean (dashed horizontal lines) and median (solid horizontal lines) of the Human Orientation Index (HOI) of the chimpanzees housed in Leintal zoo and Ngamba Island.

When the degree of human orientation of the chimpanzees at both facilities was compared (HOI), it was found that there were significant differences between the chimpanzees at both facilities, with the chimpanzees housed at Ngamba Island Sanctuary being more human oriented than those housed at Leintal Zoo (Model 3; likelihood ratio test between full and reduced model: *X*^2^ = 5.47, *df* = 1, *p* = 0.019; *R*^2^ = 0.14, Fig. 4, Table 5). When these differences were explored further in two post-hoc analyses to determine in which conditions did the chimpanzees from the two facilities differ, no effect of facility was found on the combined score of condition 1 and 2 (no food visible) (Model 3.1: likelihood ratio test between full and reduced model: *X*^2^ = 0.36, *df* = 1, *p* = 0.55; *R*^2^ = 0.05, Table 6) and a tendency was found on condition 3 (food visible) (Model 3.2: likelihood ratio test between full and reduced model: *X*^2^ = 3.51, *df* = 1, *p* = 0.061; *R*^2^ = 0.11, Table 7).

**Table 4:** Fixed effects model estimates, standard errors, degrees of freedom and *p* values of Model 2.

	Estimate	SE	df	*P*
Intercept	5.03	0.70	^a^	^a^
Facility^b^	3.43	0.78	1	<0.001
Sex^c^	−0.27	0.71	1	0.71
Age^d^	−0.44	0.34	1	0.21

**Notes.**

a Not shown because of having limited interpretation.

b Ngamba Island was the reference category.

c Male was the reference category.

d Age was z-transformed to a mean of 0 and standard deviation of 1. The mean of the original variable was 20.20 and the standard deviation was 7.78.

### Effects of arrival age and lifetime spent in the sanctuary

**Figure 5:**
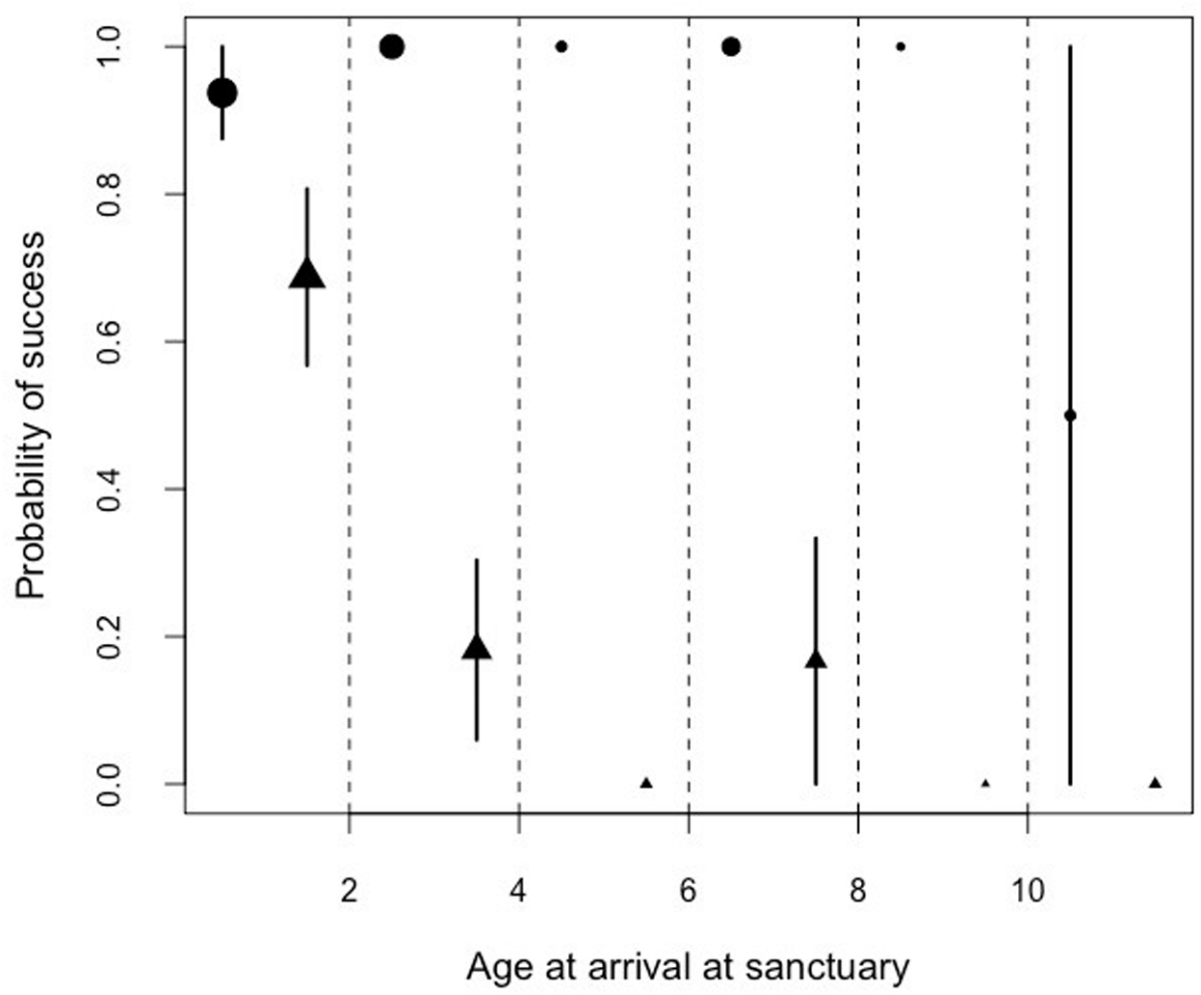
Mean probability of success and SE in the honey trap task with a stick (circles) and rope (triangle) depending on the age of the chimpanzees when they arrived at the sanctuary. The size of the points represents sample size (from left to right: *N* = 16, 11, 2, 6, 1, 2).

By examining the performance in the honey trap task in the facility with the largest sample size (Ngamba Island sanctuary), we found a significant interaction between the age of arrival at the Ngamba Sanctuary and the performance in the honey trap tasks. Performance in the honey trap varied between sub-tasks (solved with a stick or a rope) depending on the age at which the chimpanzees had arrived at Ngamba Island Sanctuary (Model 4; likelihood ratio test between full and reduced model: *X*^2^ = 64.76, *df* = 3, *p* = 0.001; *R*^2^ = 0.15, Table 8; Fig. 5).

**Table 5:** Fixed effects model estimates, standard errors, degrees of freedom and *p* values of Model 3.

	Estimate	SE	df	*P*
Facility^a^	1.23	0.54	1	0.02
Sex^b^	0.43	0.49	1	0.38
Age^c^	0.49	0.24	1	0.05

**Notes.**

a Ngamba Island was the reference category.

b Male was the reference category.

c Age was z-transformed to a mean of 0 and standard deviation of 1. The mean of the original variable was 20.48 and the standard deviation was 7.93.

**Table 6:** Fixed effects model estimates, standard errors, degrees of freedom and *p* values of Model 3.1.

	Estimate	SE	df	*P*
Facility^a^	0.31	0.52	1	0.55
Sex^b^	−0.31	0.48	1	0.52
Age^c^	0.39	0.24	1	0.10

**Notes.**

a Mother reared was the reference category.

b Male was the reference category.

c Age was z-transformed to a mean of 0 and standard deviation of 1. The mean of the original variable was 20.48 and the standard deviation was 7.93.

**Table 7:** Fixed effects model estimates, standard errors, degrees of freedom and *p* values of Model 3.2.

	Estimate	SE	df	*P*
Facility^a^	1.04	0.56	1	0.06
Sex^b^	0.57	0.52	1	0.28
Age^c^	0.39	0.24	1	0.13

**Notes.**

a Mother reared was the reference category.

b Male was the reference category.

c Age was z-transformed to a mean of 0 and standard deviation of 1. The mean of the original variable was 20.48 and the standard deviation was 7.93.

**Figure 6:**
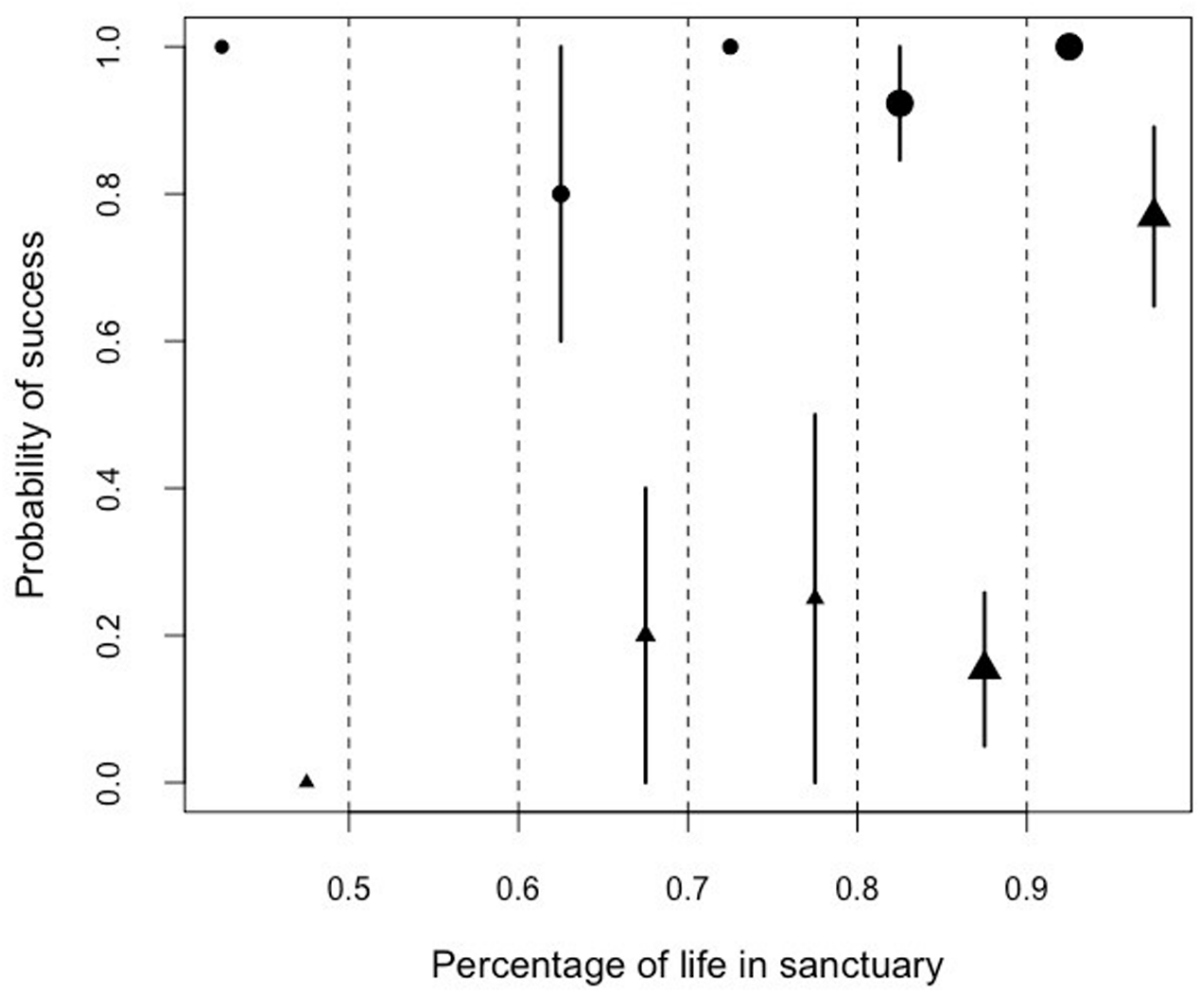
Mean probability of success and SE in the honey trap task with a stick (circles) and rope (triangle) depending on the percentage of their lives the chimpanzees had been in the sanctuary. The size of the points represents sample size (from left to right: *N* = 3, 0, 5, 4, 13, 13).

Similarly, we found that there was a significant interaction between the proportion of lifetime spent in the sanctuary and the performance in the honey trap task. Problem-solving skills varied between the two sub-tasks (solved with a stick or a rope) depending on the percentage of the life a chimpanzee had lived at Ngamba Island Sanctuary (Model 5; likelihood ratio test between full and reduced model: *X*^2^ = 65.21, *df* = 3, *p* = 0.001; *R*^2^ = 0.13, Table 9; Fig. 6).

**Table 8:** Fixed effects model estimates, standard errors, degrees of freedom and p values of Model 4.

	Estimate	SE	df	*P*
Intercept	24.744	8.209	^a^	^a^
Age^b^	1.078	5.025	1	0.83
Age at arrival at the sanctuary^c^	−5.723	4.643	1	0.217
Honey trap task^d^	−47.517	12.875	1	<0.001
Interaction between percentage of life and honey trap task	−33.985	9.875	2	<0.001

**Notes.**

a Not shown because of having limited interpretation.

b Age was z-transformed to a mean of 0 and standard deviation of 1. The mean of the original variable was 20.62 and the standard deviation was 7.96.

c Age at arrival at the sanctuary was z-transformed to a mean of 0 and standard deviation of 1. The mean of the original variable was 3.82 and the standard deviation was 3.14.

d Honey trap with stick was the reference category.

## Discussion

We found overall differences in cognitive performance between two populations of captive chimpanzees housed at a sanctuary and a zoo, with the sanctuary chimpanzees showing an average probability of success across tasks significantly higher than the zoo housed chimpanzees. We observed that in four out of the six cognitive measurements, there was intraspecific variation in problem-solving skills. However, in the reversal learning (in one of which), we did not find observable differences between facilities, even if the zoo chimpanzees had a higher average probability of success than the sanctuary chimpanzees at the first stage of the task (associative learning). In addition, we also found that within the same facility, chimpanzees differed in tool use skills, with those individuals that had arrived to the sanctuary at an earlier age and thereby spent a longer proportion of their lives in captivity being better at flexibly using tools than those individuals who arrived at a later developmental stage and thereby spent a shorter proportion of their lifetime in captivity.

**Table 9:** Fixed effects model estimates, standard errors, degrees of freedom and *p* values of Model 5.

	Estimate	SE	df	*P*
Intercept	25.890	9.309	^a^	^a^
Age^b^	−5.189	5.464	1	0.342
Percentage of life spent in the sanctuary^c^	3.853	7.611	1	0.612
Honey trap task^d^	−51.352	17.535	1	0.003
Interaction between percentage of life and honey trap task	36.704	15.512	2	0.018

**Notes.**

a Not shown because of having limited interpretation.

b Age was z-transformed to a mean of 0 and standard deviation of 1. The mean of the original variable was 20.62 and the standard deviation was 7.96.

c Percentage of life in the sanctuary was z-transformed to a mean of 0 and standard deviation of 1. The mean of the original variable was 0.80 and the standard deviation was 0.15.

d Honey trap with stick was the reference category.

### Problem-solving differences between captive facilities

We found that chimpanzees from the Ngamba Island sanctuary had on average a higher probability of success than their conspecifics housed in a Leintal zoo in the detour reaching and honey trap task (Table 1, Fig. 2). Similarly, prior research applying the Primate Cognitive Test Battery (PCTB) to sanctuary and zoo chimpanzees as well as bonobos (*Pan paniscus*), found that sanctuary apes showed similar, or improved cognitive skills compared to zoo housed conspecifics (Wobber & Hare, 2011). Previous findings on tool use skills of captive apes suggest that the intensity of human contact can impact tool use understanding in chimpanzees (Furlong, Boose & Boysen, 2008) and increase curiosity in orangutans, which in turn can lead them to improve their tool-use skills (Damerius et al., 2017a). Most chimpanzees at Ngamba Island sanctuary are orphans due to poaching, habitat destruction, or maternal death and went through years of rehabilitation. Consequently, these chimpanzees were raised and cared for by humans with extensive and close human exposure. One possible factor contributing to the observed variation in task performance between the two facilities could be the different degree of exposure to humans experienced by the chimpanzees at the zoo and the sanctuary. At Ngamba Island volunteers and tourists, who are visiting and supporting the wildlife sanctuary, can participate in chimpanzee feeding routines under the supervision of staff members. Therefore, chimpanzees at Ngamba sanctuary are often exposed to different unfamiliar humans in relatively close contact. Chimpanzees at Leintal zoo on the other hand, are only fed by their familiar caretakers and thus experience less diversity and frequency of humans closely interacting with them during feeding times. These differences in exposure to unfamiliar humans during feeding times might explain why we found differences between facilities in the degree of human orientation in the third condition of the HOI (where an unfamiliar man offered the subjects food, Model 3.2).

Between-facility differences in task performance could also be influenced by the different rearing backgrounds of the majority of chimpanzees housed at the sanctuary and the zoo. Most of the chimpanzees housed at Leintal zoo had been raised by their own mothers, in a large group of conspecifics, representing a developmental socio-environment closer to that of natural chimpanzee communities than that present in Ngamba sanctuary. Such mother-reared individuals may be less influenced by human exposure (being less human oriented, Fig. 4) despite living in a captive setting than individuals that have lost their mother and thereby have been mainly hand-reared by humans. However, it is important to note that in the zoo sample of our study, the four chimpanzees that were hand-raised by humans did not solve the more demanding tasks of detour reaching without trial and error exploration and did not use the rope tool to extract honey. Such cases suggest that, in our sample, housing facility and prior exposure to cognitive testing might have a stronger effect on task performance than rearing background. Unfortunately, given the structure of our data, it was not possible to disentangle the individual contributions of rearing background and facility to task performance. Therefore, we encourage future research to investigate the potential role of rearing background during the first years of life on multiple measures of cognitive performance. Likewise, it would be important to disentangle how various factors, such as group compositions, contact with caretakers and diet routines, at different housing facilities affects cognitive development and other behavioral outcomes (Novak et al., 2014). Other factors that could potentially contribute to the observed variation in task performance between facilities are the different levels of familiarity of the chimpanzees with the presented materials and their overall past experience in cognitive tests. The Ngamba Island chimpanzees were more used to being separated from their social group than the chimpanzees at Leintal zoo, which could explain why these chimpanzees showed higher interaction times with the honey trap test apparatus compared to the Leintal chimpanzees (Fig. 3). This higher *motivation* to manipulate and explore the apparatus could explain why they were more successful at innovating the use of the rope as a tool in this task. Recent findings from avian cognition have shown that Goffin’s cockatoos (*Cacatua goffiniana*) that were temporarily held captive were less motivated to interact with a problem-solving task than those birds that had been hand-raised and lived longer in captivity (Rössler et al., 2020). Together with our study, such results suggest that captivity and human exposure may influence the motivational level rather than the animals’ cognitive capacity per se.

The apparatus used in the detour reaching task was entirely made of Plexiglas (Fig. 1A) and previous studies have shown that orangutans with different rearing experiences vary in their problem-solving skills when presented with human-made materials like Plexiglas (Damerius et al., 2017b). The Ngamba Island chimpanzee sanctuary has hosted multiple cognitive research projects, some of which have focused on cognitive performance by presenting the chimpanzees with different types of test apparatuses including transparent materials (e.g., Horner & Whiten, 2005; Horner & Whiten, 2007; Marshall-Pescini & Whiten, 2008; Tennie, Call & Tomasello, 2010; Bullinger, Melis & Tomasello, 2014). Therefore, the Ngamba chimpanzees had previous experience with Plexiglass-like objects at the time of this study. The Leintal zoo chimpanzees on the other hand, have no previous experience with test apparatuses made from Plexiglas (and hardly any experience at all with other testing materials). Since previous experience with certain human-made materials can influence an individual’s reaction and perception of the test apparatus, the chimpanzees less familiar with these materials may need to explore the box more thoroughly before finding a solution. Accordingly, in our experiments, the differences between chimpanzees at Ngamba Island sanctuary and Leintal zoo were stronger when we examined subjects that solved the detour-reaching task *without* any prior exploration compared to when they solved the task through physical exploration (Fig. 2).

In the reversal learning task, the chimpanzees at Leintal zoo had higher average success probabilities than the sanctuary chimpanzees. This difference was likely driven by the fact that the zoo chimpanzees did better in the first part of the task (learning color association). The success in the second part of the task (reversal learning) was similar in both facilities. Since reversal learning directly depends on how fast an individual learns the first part of the task, one would have expected the zoo chimpanzees to perform better than the sanctuary chimpanzees, as their likelihood of success in the first step of the task was higher than that of sanctuary chimpanzees. However, overall success in the task was low and only 33% of all tested chimpanzees learned the reverse pattern (Table 1). Even though primates’ success in reversal learning tasks has been associated with increased brain size (Deaner et al., 2007), a comparative study across taxa reported that cleaner fish (*Labroides dimidiatus*) outperformed three primate species (Capuchin monkeys, *Cebus apella*; orangutans, *Pongo spp*; chimpanzees, *Pan troglodytes*) in reversal learning tasks (Salwiczek et al., 2012). The superior reversal learning skills of this fish species compared to the primates are arguably the consequence of the species-specific foraging ecology involving unpredictable social interactions with reef fish. In the same study, capuchins outperformed apes in reversal learning tasks (Salwiczek et al., 2012). Reversal learning tasks require a high inhibitory control and as opening the different lids in our study did not impose any direct cost for the chimpanzees, they may not have been motivated enough to inhibit incorrect responses, which could explain why the tested chimpanzees performed particularly poorly in this task.

### Tool use differences within the sanctuary

Due to the large sample size in Ngamba, we were able to examine variation in tool use skills while keeping facility constant. Comparing chimpanzees *within* the Ngamba Island sanctuary revealed that it was harder for the chimpanzees to innovate the rope solution than using the stick in the honey trap task (Table 1). The higher success rate of the stick solution could be due to the generalized use of sticks for varied foraging activities across chimpanzee populations (Whiten et al., 1999; Koops, Furuichi & Hashimoto, 2015) –i.e., to a higher likelihood of using sticks than rope as a latent solution in chimpanzees (Tennie, Call & Tomasello, 2009).

Orphans that had arrived at the sanctuary younger than two years old had the highest probability of innovating the rope solution (Fig. 5). We also found that the proportion of lifetime spent at the sanctuary had a significant influence in the success probability in the honey trap task (Fig. 6). Individuals that had spent over 90% of their lives at the sanctuary were more likely to innovate the rope solution. These results suggest that just like in human foster children (Nelson et al., 2007), the arrival age of an infant chimpanzee at the place of care (here the sanctuary) can have an impact on its cognitive skills. Given that the development of chimpanzee brain structure can be influenced by early rearing (Bogart et al., 2014), varying rearing conditions during sensitive developmental periods in the first years of life (see overview by (Gerhardt, 2014) for human infants) could have influenced the chimpanzees’ physical cognition in the tool use task. Since orphans arriving at the sanctuary before reaching their second year (thus deprived from maternal care) performed better than later arriving orphans, physical cognitive skills (at least in this task) may not be negatively influenced by early maternal separation. Previous studies on apes have shown that social interactions with conspecifics and sufficient healthcare provided by humans can compensate for early maternal loss and reverse some of the negative effects that traumatic events can have on social cognition (Meder, 1989; Martin, 2002; Bloomsmith et al., 2006; Reimers, Schwarzenberger & Preuschoft, 2007; Wobber & Hare, 2011).

## Conclusion

Our study examined within species variation in problem-solving skills by (a) comparing two groups of chimpanzees from different facilities (sanctuary versus zoo) with identical test paradigms and (b) within the same facility by evaluating the influence of time spent in captive rehabilitation. The present study shows intraspecific variation in physical cognition and an effect of facility in the chimpanzees’ motivation to explore test apparatuses, which in turn could influence cognitive performance. Intraspecific differences between facilities in task success probabilities could also be influenced (among others) by previous experience with testing apparatuses and procedures as well as different levels of exposure to humans. Chimpanzees that arrived at an early age to the sanctuary after losing their mothers and that consequently spent large parts of their lives under human care, were better tool innovators than individuals that arrived later and spent less time in the sanctuary. Given the variation between and within housing facilities that we established empirically, researchers should be careful not to automatically extrapolate from one study population towards others without considering the subjects background histories. As such, our findings stress the importance of considering cross-facility differences in primate cognitive performance and the value of multi-institutional studies that allow for further evaluation of experience and developmental effects (Many Primates et al., 2019).

##  Supplemental Information

10.7717/peerj.10263/supp-1 Supplemental Information 1 Supplemental Figures and Tables

## Additional Information and Declarations

### Field Study Permissions

The following information was supplied relating to field study approvals (i.e., approving body and any reference numbers): Barbara Gessmann (Leintal Zoo, Schweigern, Germany) provided permission to study the chimpanzees at Leintal Zoo at Leintal Zoo.

### Data Availability

The following information was supplied regarding data availability: All data and code employed in these analyses are available at OSF: Forss, Sofia. 2020. “Fork of Physical Cognition Chimpanzees.” OSF. October 16. doi:10.17605/OSF.IO/QEYDU.
