# Supplementary material for: Chimpanzees’ (Pan troglodytes) problem-solving skills are influenced by housing facility and captive care duration
Source: PeerJ. 2020 Nov 25;8:e10263. doi: 10.7717/peerj.10263 (PMC7698692; doi:10.7717/peerj.10263)
Supplement: Supplemental Information 1 [file peerj-08-10263-s001.docx]

**Chimpanzees' (*Pan troglodytes*) problem-solving skills are influenced by housing facility and captive care duration**

Short title: Captive experience effects on chimpanzee physical cognition

*Sofia Ingrid Fredrika Forss*^1^, *Alba Motes-Rodrigo*^1^, *Christine Hrubesch*^2^ & *Claudio Tennie*^1^

^1^Department of Early Prehistory and Quaternary Ecology, Eberhard Karls University Tübingen, Tübingen, Germany

^2^Department of Anthropology, University of Zürich, Zürich, Switzerland

**Supplementary material**

**STable 1**: Information of the chimpanzees that participated in the study.

| **Chimpanzee** | **Facility** | **Sex** | **Age*** | **Category** | **Background information** |
| --- | --- | --- | --- | --- | --- |
| Africa | Ngamba Island Chimpazee Sanctuary | Female | 10.5 | orphan | Arrived as 1-year old, poor body condition |
| Asega | Ngamba Island Chimpazee Sanctuary | Male | 19 | orphan | Arrived as 1-year old, suffered from malnutrition |
| Bahati | Ngamba Island Chimpazee Sanctuary | Female | 27 | unknown |  |
| Baluku | Ngamba Island Chimpazee Sanctuary | Male | 19 | orphan | Found locked up in a case, arrived as 1 year old |
| Baron | Ngamba Island Chimpazee Sanctuary | Male | 13 | orphan | Been held 12 months in a box prior to confiscation |
| Becky | Ngamba Island Chimpazee Sanctuary | Female | 26 | unknown |  |
| Bili | Ngamba Island Chimpazee Sanctuary | Female | 19 | orphan |  |
| Bwambale | Ngamba Island Chimpazee Sanctuary | Male | 18 | orphan | lived 2 years with a family prior to confiscation |
| Cindy | Ngamba Island Chimpazee Sanctuary | Female | 24 | unknown |  |
| Cocoa | Ngamba Island Chimpazee Sanctuary | Female | 12 | unknown |  |
| Ikuru | Ngamba Island Chimpazee Sanctuary | Female | 22 | Mother-reared | up to age 4 with own mother then sanctuary |
| Indi | Ngamba Island Chimpazee Sanctuary | Male | 18 | orphan |  |
| Kalema | Ngamba Island Chimpazee Sanctuary | Male | 21 | orphan | been held by soldiers and in human household prior to arrival at sanctuary |
| Kazahukire | Ngamba Island Chimpazee Sanctuary | Female | 18 | Mother-reared | up to age 3 with own mother in the wild, then sanctuary |
| Kidogo | Ngamba Island Chimpazee Sanctuary | Female | 33 | unknown |  |
| Kikyo | Ngamba Island Chimpazee Sanctuary | Male | 13 | orphan | Good health condition at arrival |
| Kisembo | Ngamba Island Chimpazee Sanctuary | Male | 18 | orphan | Good health condition at arrival |
| Madina | Ngamba Island Chimpazee Sanctuary | Female | 11 | unknown | Poor health condition, canine teeth removed, suffered from malnutrition upon arrival |
| Mawa | Ngamba Island Chimpazee Sanctuary | Male | 21 | orphan | Hand raised, but tied with a rope when found |
| Megan | Ngamba Island Chimpazee Sanctuary | Female | 33 | orphan | Circus pet in Europe prior to arrival |
| Minni | Ngamba Island Chimpazee Sanctuary | Female | 12 | orphan | Poor health condition, teeth removed and suffered from malnutrition when confiscated |
| Nagoti | Ngamba Island Chimpazee Sanctuary | Female | 31 | unknown | Poor health condition when found |
| Nakku | Ngamba Island Chimpazee Sanctuary | Female | 16 | orphan | Held by soldiers in Kampala prior to confiscation |
| Namukisa | Ngamba Island Chimpazee Sanctuary | Female | 18 | orphan |  |
| Nani | Ngamba Island Chimpazee Sanctuary | Female | 16 | orphan | Taken care of by human |
| Natasha | Ngamba Island Chimpazee Sanctuary | Female | 27 | unknown |  |
| Ndyakira | Ngamba Island Chimpazee Sanctuary | Female | 18 | orphan |  |
| Nkuumwa | Ngamba Island Chimpazee Sanctuary | Female | 21 | orphan | Held by soldiers in Kampala prior to confiscation |
| Okech | Ngamba Island Chimpazee Sanctuary | Male | 16 | orphan | Human hand fed with bottle and cared for by national park staff members prior to confiscation |
| Pasa | Ngamba Island Chimpazee Sanctuary | Female | 18 | orphan |  |
| Rambo | Ngamba Island Chimpazee Sanctuary | Male | 13 | orphan | suffered an abnormal skin disease upon arrival |
| Robbie | Ngamba Island Chimpazee Sanctuary | Male | 31 | unknown |  |
| Rutoto | Ngamba Island Chimpazee Sanctuary | Male | 13 | orphan |  |
| Sally | Ngamba Island Chimpazee Sanctuary | Female | 26 | unknown |  |
| Sara | Ngamba Island Chimpazee Sanctuary | Female | 8 | orphan | Poor health condition upon arrival |
| Sophie | Ngamba Island Chimpazee Sanctuary | Female | 31 | unknown |  |
| Tumbo | Ngamba Island Chimpazee Sanctuary | Male | 28 | unknown | Poor health condition upon arrival |
| Umugenzi | Ngamba Island Chimpazee Sanctuary | Male | 20 | orphan | Hand fed with bottle and cared for 15 months by American woman |
| Umutama | Ngamba Island Chimpazee Sanctuary | Male | 21 | orphan | Hand nursed and cared for 15 months by American woman |
| Yoyo | Ngamba Island Chimpazee Sanctuary | Female | 18 | unknown |  |
| Bea | Leintalzoo | Female | 22 | orphan | Hand raised, lived with family for 1 year, then 1 year alone, then with conspecifics |
| Dagobert | Leintalzoo | Male | 32 | Mother-reared | |
| Espe | Leintalzoo | Female | 25 | Mother-reared | |
| Felix | Leintalzoo | Male | 25 | orphan | Hand raised, lived with family prior to entering the group of conspecifics |
| Fetz | Leintalzoo | Male | 10 | Mother-reared | |
| Garibal | Leintalzoo | Male | 20 | Mother-reared | |
| Gartenzwerg | Leintalzoo | Female | 33 | Mother-reared | |
| Girlie | Leintalzoo | Female | 12 | Mother-reared | |
| Gregor | Leintalzoo | Male | 27 | Mother-reared | |
| Heronimo | Leintalzoo | Male | 31 | Mother-reared | |
| Liederli | Leintalzoo | Female | 46 | orphan | Probably born in Africa, hand reared and previously stayed at Tierpark Gettorf, Germany |
| Lindi | Leintalzoo | Female | 20 | Mother-reared | |
| Lutz | Leintalzoo | Male | 12 | Mother-reared | |
| Max | Leintalzoo | Male | 20 | Mother-reared | |
| Panya | Leintalzoo | Female | 11 | orphan | Human hand fed with bottle, lived in family home up to 1 year then peer group |
| Para | Leintalzoo | Female | 18 | Mother-reared | |
| Pitch | Leintalzoo | Female | 20 | Mother-reared | |
| Scholzi | Leintalzoo | Female | 29 | Mother-reared | |
| Toto | Leintalzoo | Male | 36 | orphan | Human hand fed with bottle, previous owner and place of birth unknown |
| Wazlaw | Leintalzoo | Male | 26 | Mother-reared | |
| Zicklein | Leintalzoo | Female | 17 | Mother-reared | |
| Zina | Leintalzoo | Female | 12 | Mother-reared | |
| Zora | Leintalzoo | Female | 7 | Mother-reared | |


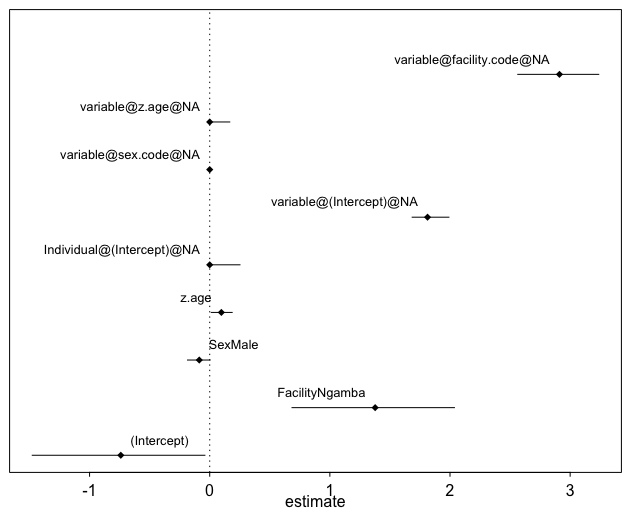


**SFigure1:** Stability plot of Model 1 produced using the minimum and maximum of the case-wise deletion of levels of random effects, together with the original estimates (diamonds). Code to produce the plot provided by Roger Mundry.


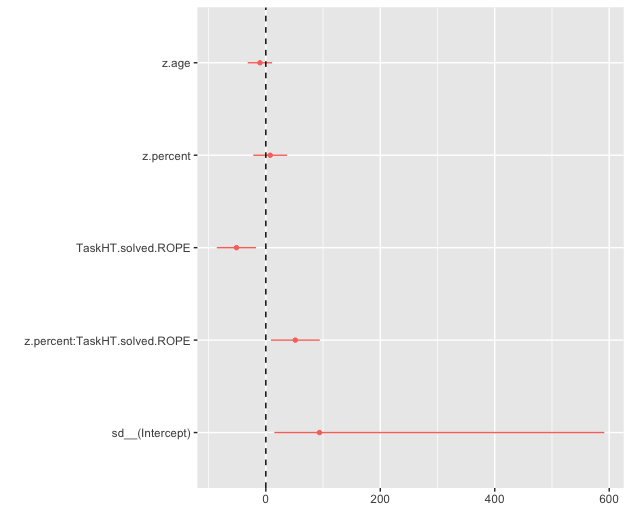


**SFigure 2**: Stability plot of Model 4 created using the packages dotwhisker and broom.mixed. Code written by Bolker (2000).


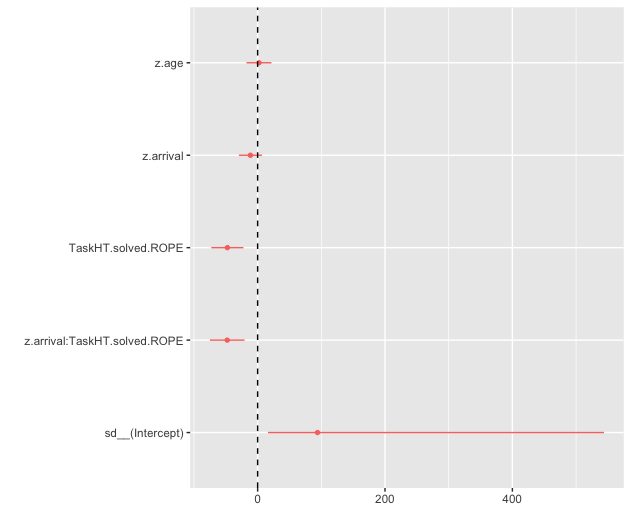


**SFigure 3**: Stability plot of Model 5 created using the packages dotwhisker and broom.mixed. Code written by Bolker (2000).

**Additional analyses 1:** **effect of rearing background on chimpanzee cognitive performance (Supplementary Model 1).** We previously analyzed the data set slightly differently and fitted separate models for each problem – solving tasks (these models can be obtained upon request by the main and second author). In these models, the response variable was the success or failure of each individual in the task. The predictor variables were facility and rearing background, which was measured in a more fine-grained manner as the variable included in the model below. However, this approach led to a very large number of models (27), which increased the risk of type II error. Furthermore, this approach did not allow disentangling the effects of the two predictor variables. Consequently, in order to increase model stability and reduce the risk of type II error we decided to focus exclusively in the facility effect (see main manuscript). However, we also wanted to explore potential differences between individuals of rearing background so we fitted a supplementary and preliminary model. This model was not included in the main manuscript because the data is very unbalanced between rearing groups. This supplementary model is identical to Model 1 except for the inclusion of rearing instead of facility. We hope that this preliminary analysis serves as a first step towards investigating differences in cognitive performance caused by different rearing backgrounds, a topic we hope future studies will pursue.

**STable 2**: Fixed effects model estimates, standard errors, degrees of freedom and p values of Supplementary Model 1.

|  | Estimate | SE | df | P |
| --- | --- | --- | --- | --- |
| Intercept | 0.96 | 0.61 | (1) | (1) |
| Rearing (2) | -1.59 | 0.54 | 1 | 0.003 |
| Sex (3) | 0.05 | 0.29 | 1 | 0.86 |
| Age (4) | 0.08 | 0.15 | 1 | 0.58 |

(1) Not shown because of having limited interpretation.

(2) Mother reared was the reference category.

(3) Male was the reference category.

(4) Age was z-transformed to a mean of 0 and standard deviation of 1. The mean of the original variable was 20.75 and the standard deviation was 7.82.

**STable 3**: BLUPs of the random effects of supplementary model 1.

|  | Intercept | Facility (1) |
| --- | --- | --- |
| Detour task | 1.27 | -0.82 |
| Detour task without exploration | -0.84 | -0.06 |
| Honey trap task solved with stick | 1.36 | -0.09 |
| Honey trap task solved with rope | -1.69 | -0.50 |
| Learn association | 0.99 | 1.21 |
| Learn reverse association | -1.25 | 0.60 |

**SFigure 4:** Mean probability of success and confidence intervals across tasks of the human-reared and mother-reared chimpanzees housed at Leintal Zoo and Ngamba Island Sanctuary. The area of the dots represents sample size. The comparison of the full model with a reduced model excluding the random slope of rearing background within task was non significant (likelihood ratio test between full and reduced model: X^2^=21.37, df=1, p<0.001).


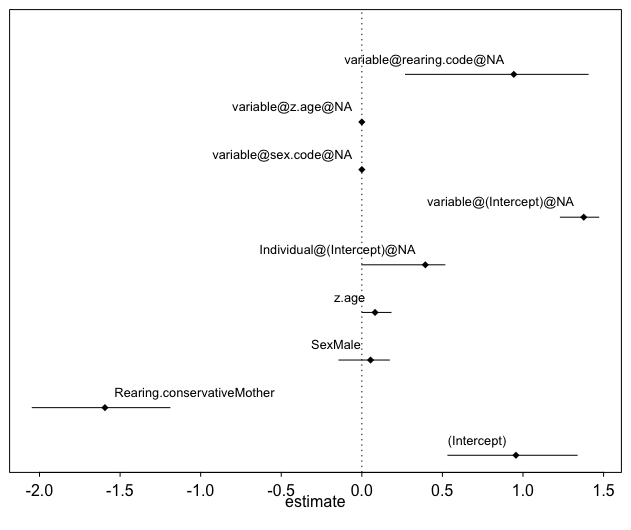


**SFigure 5**: Stability plot of Supplementary Model 1 produced using the minimum and maximum of the case-wise deletion of levels of random effects, together with the original estimates (diamonds). Code to produce the plot provided by Roger Mundry.
